# Populations Collapses in Marine Invertebrates Due to Endocrine Disruption: A Cause for Concern?

**DOI:** 10.3389/fendo.2019.00721

**Published:** 2019-10-29

**Authors:** Marcos Antonio Fernandez

**Affiliations:** Marine Ecotoxicology Laboratory, Chemical Oceanography Department, Faculty of Oceanography, Rio de Janeiro State University, Rio de Janeiro, Brazil

**Keywords:** endocrine disruption, marine invertebrates, ecological risk assessment, reproduction, environmental pollution

## Abstract

In the beginning of the twenty first century, the International Program on Chemical Safety published a document entitled *Global Assessment of the State-Of-The-Science of Endocrine Disruptors*. The work indicated only weak evidence of endocrine-related effects in human populations, and in wild animal populations. This document was revised in 2012 (*State of the Science of Endocrine Disrupting Chemicals*−*2012*) (1). The new document and the extensive scientific evidence it provided showed clearly that ED effects could be a risk to human and wildlife health. These works, however, were focused in human health and related animal models, mainly vertebrates and particularly mammals. It can be argued that invertebrates and many other taxa are important parts of all ecosystems, and, in many instances, have been shown to be also vulnerable to endocrine disruption. Thus, this work is aimed to show some observations on important marine invertebrate taxa, from an ecological point of view. The most important example of endocrine disruption in marine wild populations is the imposex response of marine gastropods, known for more than 40 years, and worldwide used to evaluate marine antifouling pollution. Among the mollusks, other important natural resources are bivalve species, used as human food sources and cephalopods, free-living, highly specialized mollusks, and also human food sources. Effects derived from endocrine disruptors in these species indicate that consumption could bring these compounds to human populations in an almost direct way, sometimes without any form of cooking or preparation. While discussing these questions, this work is also aimed to stimulate research on endocrine disruption among the invertebrate taxa that inhabited our oceans, and on which these effects are poorly known today.

## Introduction

In the beginning of the twenty first century, the International Program on Chemical Safety (IPCS, a joint program with WHO—World Health Organization and UNEP—United Nations Environment Program and the International Labor Organization) published a document entitled *Global Assessment of the State-Of-The-Science of Endocrine Disruptors* (2). This work reunited the then available scientific information on endocrine disruption (ED). The results were indicative, not conclusive: it showed that some effects observed in wildlife could be attributed to chemical compounds that can act as endocrine disruptor chemicals (EDCs), but the causal links are weak and effects related to highly polluted areas in most cases. Furthermore, the results indicated only weak evidence of endocrine-related effects in human populations. Among the studied compounds, most are POPs such as polychlorinated biphenyls (PCBs), dioxins and dichlorodiphenyltrichloroethane (DDT). The final remark was the need for broad, collaborative and international research efforts.

Against this background and putting forward a great sum of results from new research UNEP and WHO published a new document: *State of the Science of Endocrine Disrupting Chemicals*−*2012* (1). This document included three sections: the first explains the basic concepts and facts on endocrine disruption; the second discusses in detail the effects of endocrine disruptors in humans and wildlife in 12 chapters, *based in the fact that endocrine systems are very similar among vertebrate species and that endocrine effects manifest themselves independently of species*. This is an important remark for the further sections. The third and final section discusses exposure of humans and wildlife to EDCs and to potential EDCs. The key concerns derived from this impressive study are briefly showed below, as the original document is available at the WHO site (http://www.who.int/ceh/publications/endocrine/en/).

- Human and wildlife are dependent on the ability to reproduce and develop normally, what is not possible without a healthy endocrine system.
- Three evidence lines indicate concern on endocrine disruption: (i) high incidence and increasing trends of endocrine-related disorders in humans; (ii) the observation of endocrine disruption related effects in wildlife populations; (iii) identification of EDCs related to disease outcomes in laboratory studies.
- About 800 compounds are known or suspect to be able to affect: (i) hormone receptors, (ii) hormone synthesis, or, (iii) hormone conversion. Only a small fraction was thoroughly investigated. *The vast majority of chemicals in commercial use have not been tested at all*.
- Humans and wildlife are exposed to EDCs worldwide, and to more compounds than those that are POPs. However, there have been a failure in addressing the environmental causes to the increase of EDCs effects.
- The speed of disease incidence increases rules out genetic factors only as an explanation, indicating in the other hand environmental and non-genetic factors, as nutrition, exposition, and so on.
- There are critical exposure windows in the organism's development, such as fetal development or puberty, in which they are more susceptible to EDCs.
- Wildlife populations of different taxa have been affected by EDCs. In some instances, these EDCs were recognized as POPs, and *bans on these compounds have led to population's recovery* (a key remark for ecological risk evaluation).
- Internationally agreed and validated protocols to the identification of EDCs still may detect only a part of the known spectrum of ED effects. *This increases the likelihood of overlooking harmful effects in humans and wildlife*. Thus, *disease risk* (and ecological risk) *related to EDCs may be significantly underestimated*.

Even considering that some of these findings have been contested (3, 4), in this broad scenario the new document and the extensive scientific evidence it provided showed clearly that ED effects could be a risk to human and wildlife health, and that much effort is still required to a better understanding of these effects and to provide the measures required for avoiding this growing treat. While this study is a basic reference for those working in this field of research, this study was focused in human health and vertebrate models. Some instances of EDCs effects in invertebrate populations were indicated, and, in this case, with a focus in interference mechanisms and populations responses. The aim of this work is to advance a step further in the direction of the ecological risk evaluation state and requirements for the marine environments, from an ecotoxicological point of view. While being important for environmental health, these aspects are out of the scope of the original work.

## Endocrine Disruption in Marine Invertebrates: General Aspects of This Question

Invertebrates represent more than 95% of the known species in the animal kingdom, and large groups of these species are of ecological relevance in marine ecosystems (5–7). By 1999, compounds such as the common herbicides atrazine, simazine and Diuron, metals and organometallic compounds such as mercury, cadmium, or organotins, insecticides such as Toxaphene, DDT, or Endrin, alkylphenols such as nonylphenol or PCBs such as Aroclor 1242 or natural or synthetic vertebrate steroids such as diethylstilbestrol or testosterone were implicated in causing endocrine disruption in invertebrates (8). Evidence mounted ever since, and this kind of problem is being reported for several important groups of marine invertebrates, such as amphipods (9, 10), copepods, crabs, and hermit crabs (11, 12), barnacles (13), abalones (14), echinoderms (5), and polychaetes (8). In some species, intersexuality may include a simultaneous activity of both sexes gonads, in a true hermaphroditic condition (15) or could be induced by pollutants (14). Among decapoda and branchiopoda, intersexuality is fairly common, at typical background incidences of <1%, and many common pollutants have been shown to be capable to interfere with the hormonal responses (7, 11). While it is widely known that endocrine disruptors may play a key role in the conditions of marine invertebrate communities, it was often very difficult to make extrapolations from the results of studies did at cellular and sub-cellular levels to the individual and population levels for each tested species (10). A small compilation of some studies done in the last years with marine invertebrates can show the wide range of endocrine disrupting compounds and the variety of associated responses (Table 1, below). It should be noted that most of these studies do not focused in combined effects, a critical point in environmental monitoring.

**Table 1:** Some examples of endocrine disrupting compounds and their range of effects in marine invertebrate species.

**Compounds**	**Tested species**	**Effects**	**References**
TBT, DBT	*Mya arenaria*	Lower progesterone levels, sexual maturation delay, “F”	(16)
TBT	*Mya arenaria*	Skewed sex ratio, vitellin reduction, oestradiol-17β production in gonad reduced “F”	(17)
TBT	*Ruditapes decussata*	Increase in testosterone, oestradiol decrease “F”	(18)
TBT	*Haliotis madaka*	Intersexuality, ovary spermatogenesis, “F” **populations reduction**	(14)
North Sea Oil (NSO)	*Mytilus edulis*	Ovarian follicle development, normal spermatogenesis	(19)
NSO + PAH + Alkylphenols	*Mytilus edulis*	Male gonadal melanomacrophage centers, degeneration ovary follicles	(19)
Bisphenol A	*Mytilus edulis*	Spawning induction for both sexes, ovocyte atresia	(20)
2,2′,4,4′-tetrabromodiphenyl ether	*Mytilus edulis*	Ovocyte atresia, male spawning induction	(20)
Diallyl phthalate	*Mytilus edulis*	Follicle and ovocyte reduction, male spawning induction	(20)
PAHs, TBT	*Mytilus galloprovincialis*	Intersexuality, oocite atresia “F”	(21)
Benzo(a)pyrene	*Portunus trituberculatus*	Reduced ovarian growth, testosterone, progesterone and 17βestradiol secretion reduction	(22)
Benzo(a)pyrene	*Chlamys farreri*	Reduced testosterone and 17βestradiol production, progesterone disruption in ovary, ovarian impairment, development delay	(23)
Bisphenol A, 17βestradiol	*Mytilus galloprovincialis*	Gene transcription	(24)
Testosterone	*Brachionus calyciflorus*	Increased swimming, fertilization rate and recognition ability in males	(25)
Progesterone, flutamide (non-steroidal anti-androgen)	*Brachionus calyciflorus*	Inhibited swimming speed, suppression of fertilization and reduced recognition ability in males	(25)
Dibutyl phthalate	*Galeolaria caespitosa*	Sperm dysfunction, impaired embryogenesis	(26)
Propylparaben	*Tigriopus japonicus*	Sex ratio alteration toward females	(27)

*Most were laboratory studies, while those including field studies are indicated by the letter “F.” Bold letters, population reduction observed*.

Even as marked changes in marine invertebrate populations in some instances where demonstrated to occur, mainly molluscan populations, whole ecosystem, multitaxonomic environmental monitoring is seldom possible due to technical and funding questions. Only in some limited instances specific populations' distributions data are available, and mostly related to monitoring species, or those of great economic value (28). In many instances of toxicity assessment, single invertebrate species are being used to perform toxicity tests to evaluate potential responses of organisms of many different phyla, as pointed out by Depledge and Billinghurst (8). However, 20 years later this approach is still being used in many, if not most, instances.

In regard of population dynamics, some very important gaps in the available knowledge about environmental effects of pollutants are still present, turning the integration of ecologic and ecotoxicologic information even more difficult. Aspects such as habitat loss due to growing human pressure (29), the lack of specific knowledge of invertebrate endocrine systems, that are very different from vertebrate ones (6, 13), the assimilation pathways of pollutants such as water exposition, dietary exposition, feeding habits (8, 29) and also the very important questions of species responses at different development phases of the reproductive cycle and to mixtures of pollutants which may show similar/dissimilar effects (30). When the great variability of natural processes in marine communities is taken in account, it is not difficult to understand why seldom population's declines in marine invertebrates' communities have been shown to be derived from external forcing such as pollutant pressure. In the particular case of endocrine disruptors, the relative potency of each compound for the studied species is badly known, what makes the evaluation of combined toxicities a still more uncertain affair (6, 29).

As a concluding remark for this introducing section, I would argue that in the case of invertebrates the most impacting effects of pollutants, including endocrine disruptors, are those that could be strongly related to the occurrence of known pollutants affecting and, in some instances, eradicating or seriously compromising natural populations, and thus, affecting the marine ecosystems from an ecological point of view, or, also, compromising biological productivity. The most striking study cases are those related to molluscan species, and these will be the focus of the next sections.

Population decline, local extinctions or reduced reproductive capacity have been demonstrated to be directly related to endocrine disruption in three different conditions: the worldwide development of imposex in marine gastropod species (31), the occurrence of intersexuality in the abalone in Japan, which also led to some documented population reduction caused by reproductive failure (14) and the case of the Basin d'Arcachon, where bivalve commercial production collapsed during the peak of organotins application as biocides in marine antifouling paints and subsequently recovered as this application was restricted, France being the first country to exert this control (32, 33). As marine bivalves and cephalopods are also part of human diet in coastal areas (34), they are further discussed.

## Endocrine Disruption: Detection and Evaluation of Effects in Marine Gastropod Populations in a Low Organotins Exposition Scenario

In respect to ED in wildlife marine invertebrate populations, the most characteristic phenomena in organotins polluted areas is a syndrome that is called “imposex” in female gastropods. This syndrome consists in the imposition of male sexual characters, such as penis and/or vas deferens in female individuals. Smith (35), introduced this term after reports of a “penis like” growth of tissue behind the right tentacle of female gastropods, in the location of the male penis. Further research indicated the antifouling biocide tributyltin (TBT), then in intensive application in any kind of vessel as the main cause, and intensive boat and shipping activities areas as the most affected ones (36–39). By 1991, this problem had been reported in 132 gastropod species, this number rising to 192 by 2005 (40). The last extensive report raised again this figure to 268 species by 2009 (41). In the other hand, these last authors indicated some 42 gastropod species that does not develop masculinization when exposed to this compound. Species differential sensitivity, phylogeny—mesogastropods are remarkably less sensitive than neogastropods—and feeding habits are possible causes for these observations. There are many theories to explain the occurrence of this kind of DE syndrome, but the complete mechanism has not yet been totally explained (6, 41, 42).

However, as reports mounted in the literature, the problem of TBT antifoulings pollution was perceived to be global, as first indicated by Ellis and Pattisina (31). Many techniques were developed for imposex evaluation when field and laboratorial studies showed that the relative development of masculine characters on the females was dose-dependent for TBT and in some species also to TPT (triphenyltin, an alternative for TBT as biocide in the paints formulation). Development of these techniques resulted in the application of imposex development indexes, other than the simpler evaluation of the percentage of affected females in each given sampled population. These indexes were based on two approaches: those that compared the penis development in males and affected females, and those that followed the development of the vas deferens in affected females. In the first case, these indexes are the Relative Penis Length Index (RPLI) or the Relative Penis Size Index (RPSI) [(43), for a full description of measurements and application]. In the second case, the index is the Vas Deferens Sequence Index or VDSI [please refer to (40, 43–45) for particular applications of this approach]. These techniques provided the researchers with means to evaluate the relative intensity of the pollution and the extension of the affected areas with a very simple and cost effective monitoring tool.

Obviously, the ideal case is to have parallel chemical analysis for this monitoring, being these analyses of water (44), of sediments (46), or of the animals tissues (47). In the most ideal case, the intensity of imposex in gastropod populations or the organotin body burden of the animals could have provided a proxy of mean TBT water concentrations (44), but the environmental variability is such that these approaches were never thoroughly developed. Another combined monitoring approach, using imposex in gastropods populations to guide sediments sampling to the more critical areas is of easier application. Persistence of organotin pollution in conditions such as fine-grained, organic-rich, mostly anoxic coastal sediments (48, 49) has made TBT and other organotins legacy pollutants, being considered as POPs by WHO-UNEP (1). As a matter of fact, this is probably the most important reason that would explain why imposex is still being reported in European waters (50–53), even when clear instances of improvement are being reported (54). The same occurs in other areas were organotins uses were banned, such as Korea, for instance (55, 56). In the other hand, unregulated use of these compounds have been already demonstrated in some areas, for instance, Latin America (57–59) or North Africa (60–62).

**Figure 1:**
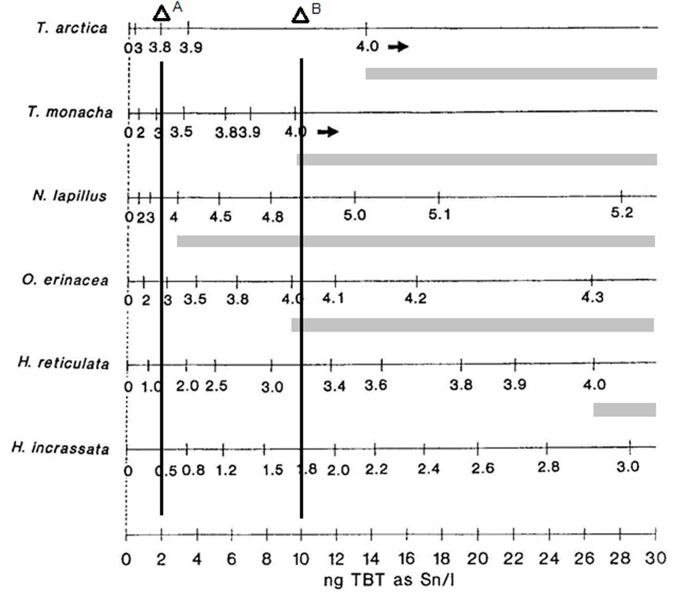
Imposex development is different marine gastropod species at increasing water TBT concentrations. Imposex intensity: values of VDSI index measured for each species; TBT water concentrations in ng(Sn). L^−1^. Ecological risk indicated by light gray bars indicating population damage by lack of recruitment due to female sterility. Vertical concentration lines: A: TBT EQS of the UK; B: Brazilian limit for sea waters, Class 1, CONAMA Resolution 357. Modified from Stroben et al. (44) by the author.

From an ecotoxicological point of view, the work of Stroben et al. (44), being multispecific, clearly demonstrated that species sensitivity could be different even in the same genus, and thus, indicated that antifouling pollution could affect marine communities as a selective pressure. In Figure 1, below, some results of this study are presented and discussed.

**Figure 2:**
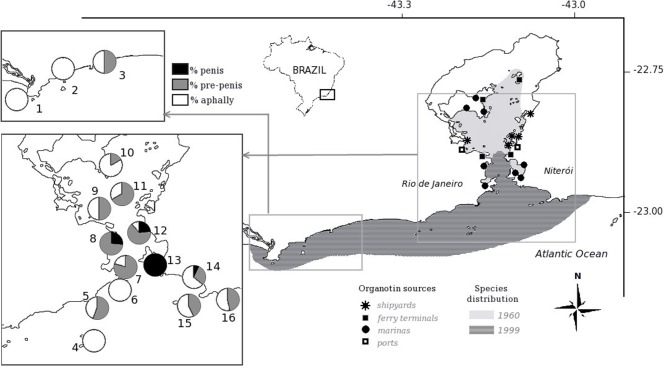
Evolution of the marine gastropod *Stramonita brasiliensis* populations at Guanabara Bay, Brazil, between 1960 and 2013. Organotin sources and expression of imposex development are also shown. Modified from Toste et al. (45).

As we can see from the original data, the species *Nucella lapillus* and *Ocenebra erinacea* are much more sensible, presenting a much more developed vas deferens than *Trivia* species or *Hinia* species at a given TBT concentration. Thus, exposed to similar conditions, the pollution effects on individuals and populations will differ greatly among the different species present at each site. Surely, not all species sampled occurred at all places at the same time. In any case, at the UK environmental target concentration of 2 ng(Sn). L^−1^ (vertical line 1), for instance, the four most sensitive species will present imposex, while both less sensitive *Hinia* species will not. All tested species that present imposex in this concentration range will have females presenting a small penis and/or a partially developed vas deferens, but no sterile individuals in the populations that reach these VDSI values (please refer to the original work for the details of each species VDSI development evaluation). However, at the 10 ng(Sn). L^−1^ concentration level, vertical line 2, that was then fairly common in coastal waters, the more sensitive species VDSI values would be above stage 4, indicating that populations began to show sterile females and thus were in danger by recruitment reduction. Local extinction of the most sensitive species was observed all around the world, sometimes eradicating part of the previous species population's distributions. For instance, some two thirds of the *Stramonita brasiliensis* populations area in Guanabara Bay, a highly polluted harbor area in Brazil, were lost between the sixties and the nineties (46). About half of this area was recovered by this species by 2012 [(45); see the details in Figure 2 below for the area extension].

The recovery of affected populations after the controls on TBT application as biocide and further banning has being considered as a sure indication of pollution reduction (54). If only marine gastropods were affected by TBT, this would have been serious enough, but lack of knowledge of the response of other marine species to this compound makes the hypothesis of ecosystem recovery somewhat less consistent (63). Even in the case of gastropods, recent research indicated that resistance to TBT effects could control the distribution of two species with different organotin sensitivity of *Leucozonia* genus, at least in a heavily polluted, big harbor area (64).

Other important recent observations on imposex development are related to aphallic imposex expression, thus separating the two classic ways of imposex intensity evaluation (by females' penis lengths or by vas deferens development). Aphally in marine gastropods was first showed to occur in *Nucella lapillus* males, in a specific location in England, Dumpton Gap. This syndrome was reported as a genetic problem that caused male specimens to have undeveloped sexual characters, malformations or even to lack their penises. In the other hand, the syndrome caused a reduction in imposex development in the females, thus permitting an isolated population to survive in a heavily polluted coast. This particular condition was called “Dumpton syndrome” because it was discovered at Dumpton Gap (65, 66). By the late nineties, this syndrome has been described in Brittany (67, 68) and in the northwest coast of Spain (69). These last authors proposed a modified VDSI evaluation scheme, as sterile females *Nucella lapillus* were observed for the first time lacking penises. This observation made clear that penis development in imposex females may be independent of vas deferens development. Because of this observation, the authors pointed that in DS conditions, or, for instance, *at lower ambient organotins concentrations*, the VDSI so modified would be a better indicator of TBT pollution that indexes such as the RPLI or RPSI that would be meaningless for aphallic females. More recently, and under different experimental conditions, it was demonstrated that TPT (triphenyltin), a tri-substituted organotin used as biocide as substitute for TBT when this compound began to be controlled, mainly in Japan (70, 71) could induce aphallic imposex development in the same species (72). While these studies were related to *N. lapillus*, aphally is recorded in other gastropod species.

In the previously quoted work by Stroben et al. (44), the species *N. lapillus, Trivia arctica, T. monacha*, and *Hinia reticulata* were showed to present complete vas deferens development from near the base of the right tentacle, where the penis is located in the males, to the vulva opening, without penis development. In *Cantharus cecillei*, this same general development pattern was observed to occur, while presenting some specific differences [see (40), for the complete observations]. In these species, however, no observation was made about *male* aphally, what would indicate that these ways of imposex development were not related to DS. In another series of works with *Stramonita brasiliensis* in the Brazilian coast, female aphally was frequently observed (73, 74). The proportional incidence of female aphallic imposex development was showed to be related to the distance from the organotins sources at Guanabara Bay, along a sensible distance from the main sources area (to some 60 km distance of the organotins sources centroid, Spearman test *R* = 0.6959, *p* < 0.05), with no male aphally being observed (45). Thus, it became clear that at least for *S. brasiliensis* vas deferens development is independent of that of the penis and penis development occurred only closer to the organotin sources inside Guanabara Bay, and thus, at higher environmental concentrations.

**Figure 3:**
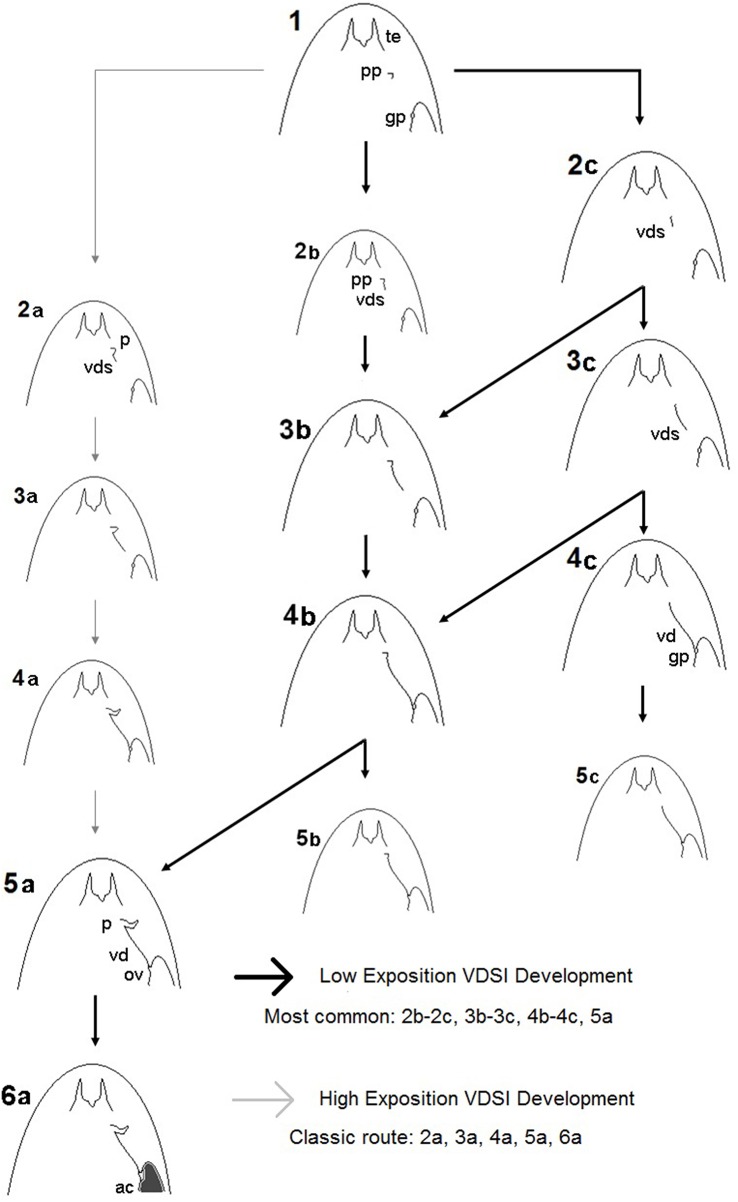
Imposex development scheme for *Stramonita brasiliana (Stramonita haemastoma as originally published)* with high and low exposition routes, according to Toste et al. (45). Captions: te, tentacle; pp, penis primordium; gp, genital papilla; vds, vas deferens; ov, obstructed vulva; ac, aborted capsules. The inclination of the routes at the same stages is related to relative organotins exposition: the nearer to stage 1, lower expositions were indicated by distance for organotins sources in the field. Dark arrows indicate the more commonly observed routes in Southeastern Brazil.

What is more important, it was observed that imposex females could even be sterilized without the development of a penis, a very important observation for environmental monitoring using imposex response that was first demonstrated by Barreiro et al. (69) in *N. lapillus*. Based in these observations, a new imposex development scheme for VDSI in *Stramonita brasiliensis* was proposed, with low and high exposition routes, that is shown in Figure 3, modified from Toste et al. (45), below. These observations could be important for other imposex monitoring studies, as aphally in imposex females have been reported in some gastropod species, such as *Hexaplex trunculus*, Lahbib et al. (75); *Stramonita rustica*, Artifon et al. (76); *Thais brevidentata, Thais bisserialis, Thais kiosquiformis, Thais melones, Plicopurpura pansa, Plicopurpura columellaris*, Grimón et al. (77). These observations with other species confirmed that VDSI could be the only adequate approach for imposex intensity evaluation. With the global reduction of TBT pollution, worldwide demonstrated by dozens of published works of measured organotins concentrations worldwide in environmental matrices, the classic imposex evaluation approach seems to need a revision. The VDSI application as shown by Toste et al. (45), may even help discriminating higher from lower exposition conditions for the studied gastropod populations, what could be useful in evaluating the relative importance of the remaining organotins sources, or of their illegal use. This approach, however, still requires the sacrifice of the studied animals, what would not be required by the use of a non-destructive approach such as the one developed by Fernandez et al. (73).

The widespread occurrence of imposex female aphally renders penis lengths indexes meaningless (for instance, total female aphally was recorded for 9 out of 19 stations for imposex monitoring in *Nucella lapillus* in 2014, causing values for RPLI in this species along the Portuguese coast to drop to almost zero, thus rendering its application unable to further indicate decreasing tendencies afterward but for one sampling station along the Portuguese coast [(53), Table 1]. The same observation was reported in *Hexaplex trunculus*, showing a temporal reduction pattern of phallic imposex development as observed in *Stramonita brasiliensis* in Brazil, by Lahbib et al. (78) in Tunisia. The use of vas deferens development only, without taking in account the penis development, for VDSI application was even indicated for *Nucella lapillus* by (79). In the other way, some recent studies done in more heavily polluted areas that still remain in Latin America, such as Peru (59, 80) and Chile (81), for instance, showed that penis-based indexes are still useful and could still provide relevant information for some time to come in the remaining hot spots areas. Anyway, the aphallic imposex development observed for *S. brasiliensis* could lead to female sterility, as previously pointed out, when the females presented only pre-penises or very small penises (45), thus making the VDSI the most relevant imposex development index to be applied in this new, mostly low exposure scenario.

There is still another complicating factor in the field imposex analysis: the quite relevant question of interference in the imposex response of the animals. This interference may arise by two different mechanisms: one is the complexation of organotins when high loads of organic matter are present in the waters at the same time. It was long known that organic matter has as very strong affinity to organotins (82) and that in anoxic sediments, degradation of organotins is very slow (83, 84). In any case, this same high affinity will be present in waters rich in POC (particulate organic carbon) and DOC (dissolved organic carbon). These organic compounds thus may act as a kind of “buffer,” reducing the bioavailability of organotins to the biota, and, consequently, the imposex expression of gastropod populations. Frequently associated to organic rich waters in the coastal zone are direct sewage discharges, important artificial sources of POC and DOC. At the same time, these sewage discharges are also important sources of xenoestrogens to the same coastal areas. It has been demonstrated by bioassays with *Nucella lapillus* using sewage treatment plant effluents rich in xenoestrogens such as octylphenol, nonylphenol, and bisphenol A that exposition to these effluents could activate the estrogen receptor of this species (85) and at the same time were capable of reducing the imposex expression of TBT-treated females (86). The most affected response is, not unexpectedly, the RPLI, as we saw the growing relative importance of the aphallic vas deferens development routes before.

**Figure 4:**
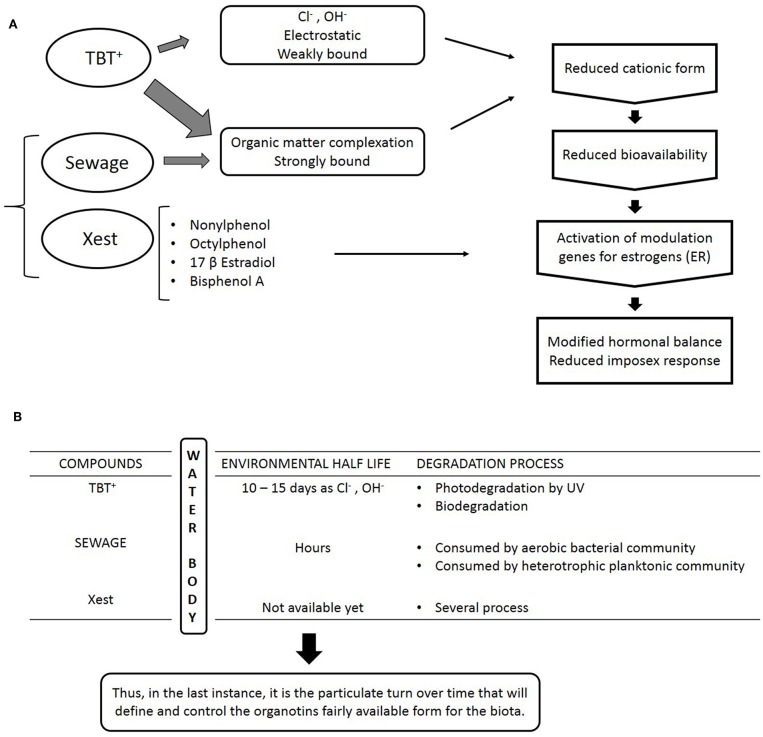
**(A)** Schematic scheme for an interference mechanism in the imposex response of marine gastropod mollusk to organotin compounds. While cationic organotins are the most available form to biota, both particulate and dissolved organic matter from sewage can complex organotins, while sewage is also a source of xenoestrogens that may act as antagonists to imposex development. **(B)** Main environmental aspects of the interference mechanism proposed. Due to the multiple variables involved, only the general lines are indicated.

This interference mechanism was observed in the field for the first time at a small touristic city, called Paraty, which is located at the end of a small inlet in the southeastern coast of Rio de Janeiro state, Brazil. Coincidently, in this area, the organotin sources and organic matter sources are located very close to each other, in the inner inlet, and this particularity made possible to understand the different water residence times of each compound. Monitoring studies of imposex development in populations of *S. brasiliensis* were made in 2006 and 2011, and while a relative amelioration was observed in the inner inlet stations, the outer stations showed a relative aggravation of the imposex condition. The most likely reasons for this strange observation are that while organotins were present in the whole area, and recent input was showed to occur at the 2011 sampling by sediment analysis, the number of small boats, that are the only sources in the area, remained approximately constant while local human population has grown. Thus, organic matter and xenoestrogens inputs, aggravated by total lack of sewage treatment, also have certainly risen. Then, the “buffer” effect was enough to suppress part of the imposex development of the animals in the inner inlet. The populations recovered, while showing 100% imposex low expression incidence (only two penis bearing females recorded in the study). In the other hand, degradation of the organic matter and dispersion reduced the “buffer” effect in the outer inlet, and thus the remaining organotins are still able to produce a response in the animals. While somewhat speculative, the key to understand this process was the difference in water residence times of organotins, that are highly toxic to marine organisms, and of the sewage derived organic matter, that is highly nutritive, and thus, quickly degraded by aerobic bacteria. While not all these parameters were measured in occasion, the very color and particulate matter content of the waters from the inner inlet when compared to those of the outer inlet showed clearly where the problem was. A schematic conceptual description of this interference mechanism is shown in Figures 4A,B, below. For the original data and details, please refer to Borges et al. (87).

Putting all these information and ideas together, what seems clear is that in a new scenario of lower organotins inputs, in many instances derived from contaminated sediments, including in Europe (48, 88–90), the imposex response of marine gastropod populations should be evaluated with care. Under conditions of interference with this response, imposex development could be reduced in some areas, mostly in urbanized areas with parallel sewage discharge pollution. In these conditions, while the animals may show a low imposex response, the animals' body burden of organotins could still be high, even higher than in more pristine conditions. Some recent results seems to indicate this possibility with animals presenting high organotins body burden with showing low imposex responses (59, 91). Some aspects of the populations used for biomonitoring have influence on the results, such as genetics, temperature influence on metabolism or even the seasonality of the reproductive cycle, a basic aspect often not considered (92). In any case, the key to understand the animal's response would be the possibility of interference by other parameters in the water, which is dependent of each study area particularities. The conclusion that organotin pollution has been ameliorated may be doubtful in some situations. As biological monitoring is frequently used without bioassays or body burden analysis, the result of these studies must be evaluated with care. If interference is suspected, confirmatory chemical analysis is indicated, and occurrence of organotin pollution should be suspected. *In this case, the very occurrence of imposex in marine snails is a clue that biologically active organotin compounds are present in local waters*. It is important to remember that organotins could present a human health risk for some coastal populations, as previously reported (34, 76, 81, 93, 94). Imposex development is still the faster and cheaper biological monitoring method to evaluate the occurrence of remaining hotspots of organotin pollution, or to verify its illegal use.

## Endocrine Disruption in Marine Bivalve Mollusks: Growing in Importance as Biomonitoring Organisms in a Near Future

Among all animal taxa, bivalve mollusks are probably the most important for monitoring the extension and intensity of marine pollution. Numerous programs of marine monitoring employed bivalve mollusks, among which the Mussel Watch was the most important. Being sessile, resistant, easily collected, these are adequate organisms for monitoring studies. The fact that bivalves are also important components of many human populations diet in coastal areas, and the object of growing mariculture investments make these species important vectors of transference of pollutants introduced in these coastal areas to human populations. Marine bivalves have also been shown to present reproductive anomalies related to endocrine disruptors. When the bivalve *Ruditapes decussatus* was transplanted to TBT polluted areas, a rising on testosterone levels with estradiol reduction was observed (18). This same observation was made on *Mya arenaria* populations (16). This last species had shown male-biased populations in another study, by Gagné et al. (17), the first instance in which hormonal alterations were reflected at population level. The occurrence of intersexuality has been showed to occur in marine bivalves (21). On the subject of endocrine disruption, the most common studies were focused in the occurrence of vitellogenin proteins in males, for instance, of *Tapes philippinarum* (95–97) or *Mytilus edulis* (19, 20, 98). This occurrence is related to availability of xenobiotics such as nonylphenol, bisphenol A, ethinylestradiol, or PAHs, common in urban sewage (99–103). In spite of many instances of endocrine disruption being observed within populations of marine bivalves, the extinction of populations was not commonly observed. From this point of view, the most studied case of extensive populational damage was the Arcachon Bay, France, and the commercial farming of *Crassostrea gigas*, the Pacific oyster.

The Basin D'Arcachon is a closed roughly triangular tidal water body with some 156 km^2^ area, in which some 10,000 to 15,000 tons of commercial oysters were produced each year. From 1975 to 1982, oyster production was severely reduced, due to absence of spatfall and anomalous growth, with shell calcification anomalies (32). While these problems were reported at the same time in other areas with this same species in England and also along the Spanish Mediterranean coast, the most important and studied area was Arcachon (104). In a very interesting review, Ruiz et al. (33), indicated that from initial water concentrations of TBT of <1 ng(Sn)L^−1^ by 1960, the increased use of this compound led these concentrations to rise above 100 ng(Sn)L^−1^ by 1981–1982, when controls on application were applied by the French government. After these measures, water concentrations of TBT decreased to about 10 ng(Sn)L^−1^ by 1987, reaching again about 1 ng(Sn)L^−1^ by 1993. These same authors have also shown that besides the oyster production collapse, other important ecological changes occurred in the same area. Simultaneously, it was observed a great reduction of the local populations of the gastropod *Ocenebra erinacea*, in which the first symptoms of imposex development were reported by 1973, earlier than the oyster production collapse. With the reduction of organotin pollution, gastropod populations recovered later. Also “green tides” of *Enteromorpha* spp. were reported to occur by 1982. While clearly indicating the gaps in the original data, the authors indicated that TBT effects on other invertebrate grazers may explain this anomalous observation, a first evidence of multispecific TBT ecological disturbance. Our research group has also noted excessive algal growth and apparent reduction of herbivorous species in some organotin polluted coastal areas in Brazil. This subject is now under a closer scrutiny, as our previous studies were designed for imposex evaluation only.

It should be pointed out, also, that dioecious bivalves did not have the internal fecundation of marine gastropods that made gastropods such useful species for environmental monitoring of endocrine disruption. Reproductive conditions evaluation in marine bivalves often requires histological analysis, what precludes a quick, fast, effective monitoring methodology such as imposex evaluation. With the phasing out of TBT, and the intensive use of new antifoulings that may act as xenoestrogens or estrogen agonists, such as Irgarol 1051, the “naval version” of atrazine [see (73) for a deeper discussion], imposex will be used only in the monitoring of the remaining hotspots of legacy organotin polluted areas. The relative importance of marine bivalves as indicators of marine endocrine disruption will probably rise in a near future, including in the evaluation of health risks for coastal human populations.

## Possible New Targets for ED Compounds: Marine Cephalopod Mollusks

Cephalopods are free-living predator mollusks with high mobility and very effective sensorial capabilities, almost completely opposed to the typical oyster species in respect of adequation for environmental monitoring studies. They are in some instances important and appreciated food items, and have been included in human risk analysis for coastal areas (34). From an endocrine disruption point of view, cephalopods have a more complex nervous system than the other mollusks. While in bivalves and gastropods neurohormones secreted by nervous ganglia and gonads are responsible for sexual maturation, showing first and second order control systems, cephalopods show a third order neuroendocrine control system that is comparable to the vertebrate HPG ax [see (105)]. In this sophisticated control system, the Octopus Gonadotrophin-Releasing Hormone (Oct-GnRH) has been shown to act as modulator in functions such as feeding, memory or sensorial, as well as in steroidogenesis in *Octopus vulgaris* (106). In the other hand, it has been shown that estradiol regulates Oct-GnRH and several functions of the nervous systems in the same species (107). As cephalopods are being considered excellent candidates for mariculture in Europe (105), a cycle may be closing on them: as xenoestrogens have been shown to be present in many instances in coastal areas and can affect these animals, their use in mariculture will turn them sessile for all environmental aspects such as bioaccumulation and pollutants transference for humans. Clearly more research is required on this subject.

## Endocrine Disruption and Ecological Risk Assessment

The global aspects of endocrine disruption may be inferred by the ubiquity of detection of proven or suspect endocrine disrupting compounds, even while the biological effects in invertebrate populations are not clearly shown yet. Simple and inexpensive monitoring tools as imposex induction was for organotins monitoring are still lacking, and tools as vitellogenin induction in males are not specific. Certainly a weight of evidence approach will be required at each location based on previous knowledge of possible ED compounds sources and loads. However, in the same way that imposex have been shown to occur worldwide, and to have caused many gastropod species local extinctions, there is no plausible reason today to suppose that the ubiquity of ED compounds is not producing population damage on many invertebrate species, damage that cannot be observed with current methods and approaches. It was recently demonstrated that male infertility could be induced by several environmental contaminants (108), and that even low incidence of female intersexuality in crustacean populations may have drastic effects at population level (10, 109).

Ecological risk assessment is one of the most difficult tasks today, because it requires a deep knowledge of at last three important fields that should be employed at the same time. These are: (i) the pollutant chemical properties and behavior in aquatic environments, that will define its speciation and reactivity; (ii) the physical components of dispersion and mixing that will also influence the compound's water residence time, which is the basic aspect to indicate the exposition of aquatic communities to pollutants: and (iii) the response of each individual species to the pollutant. This is the basic scenario for single pollutants exposition. Much of the current risk evaluation works relay on these three basic kinds of information. First, the available chemical data for each compound are not always complete or reliable. Second, the mean concentrations reported for the studied compounds in the literature were seldom based on modeled concentrations in any particular area, let alone thoroughly calibrated dispersion models (110–112). Third, the available database of biological effects of the ED tested compounds is very far from being complete.

While presenting today's best available technology, would these approaches be sufficient to understand the selective pressures posed by the actual sum of anthropogenic compounds present in coastal areas? I believe these are not. Experience has shown that as more studies developed, and as the legislations advance, still the biological communities change and productivity and biodiversity decreases, and the assessment of these changes depends on expensive ecological studies that are not usually made with the appropriate extension and frequency. To improve this situation, it would be important to advance along three separate lines of action at the same time:

Axis (i) the lack of knowledge on DE effects on invertebrates of many compounds in current use tool should be a priority in research. The importance of this point was shown in the particular study case of marine antifouling paints by interference these effects can induce in the evaluation of the most used biomonitoring approach.

Axis (ii) the problem of the “cocktail effect,” not only by the way of sheer toxicity—what means acute or extensive effects -, but by the cumulative action of pollutants on the hormonal regulation mechanisms, should be another. This line is consistent with the results on human and vertebrate health discussed by WHO-UNEP (1).

Axis iii) the final considerations on the difficulty of tracing specific compounds effects on marine communities without knowing the “cocktail” composition, based on antifoulings examples previously discussed (112). This means a necessity for stronger simultaneous determination methods for multiple target compounds, and a relative potency scale for ED compounds in multiple marine taxa.

Certainly, there are other factors on these ecological risk evaluations, such as human population growth, that is greater in the coastal areas; climatic changes; the changing uses of littoral areas; overexploitation of marine resources (113). But we should always try to control the introduction in the environment of hazardous and potentially hazardous chemicals, including known and possible endocrine disruptors.

## Final Remarks

I guess in the near future it would be required to focus on the necessity of integrated studies, and on some measures required to make these studies easier to integrate. To reach this goal, we will need a relative potency scale for EDCs in marine species, an integrated database of EDCs with padronized doses and responses easily accessible to researchers and a combined chemical-ecotoxicological-ecological modeling and monitoring approach as the desired end-point. A growing number of works is appearing studying pollutants interactions to different taxa, and these efforts should be supported, because as pointed elsewhere, the interactions are not predicable. To finish this discussion, I would like to point out that specific bioindicators for ED in fieldwork in these new times would be much more probably the exception than the rule.

## Author's Note

My idea is that through the case of marine gastropod and bivalve mollusks to raise interest in research on the ecotoxicological and ecological effects of endocrine disruptors. Among marine invertebrates, endocrine disruption could be widespread, as I tried to show with the particular cases discussed. In the same way that the effects of endocrine disruptors are still poorly known in human and vertebrate populations, invertebrates could also be at risk, as several instances of populations extinctions and recuperation have been demonstrated. So, while by the point of view of human health research is much needed in this field, in the case of ecological damage and ecosystems functions much research is still required too. Perhaps even a specific topic may be raised on this subject.

## Author Contributions

The author confirms being the sole contributor of this work and has approved it for publication.

### Conflict of Interest

The author declares that the research was conducted in the absence of any commercial or financial relationships that could be construed as a potential conflict of interest.

## Abbreviations

CONAMA: National Environmental Council of Brazil
DDT: Dichlorodiphenyltrichloroethane
DOC: Dissolved Organic Carbon
ED: Endocrine Disruption
EDC: Endocrine Disrupting Compound(s)
EQS: Environmental Quality Standard
IPCS: International Program on Chemical Safety
HPG: hypothalamic–pituitary–gonadal
Oct-GnRH: Octopus Gonadotrophin-Releasing Hormone
PAH: Polycyclic Aromatic Hydrocarbon(s)
PCB: Polychlorinated biphenyls
POP: Persistent Organic Pollutant(s)
POC: Particulate Organic Carbon
RPLI: Relative Penis Length Index
RPSI: Relative Penis Size Index
TBT: Tributyltin
TPT: Triphenyltin
UNEP: United Nations Environment Program
VDSI: Vas Deferens Sequence Index
WHO: World Health Organization.
